# Resveratrol attenuated high intensity exercise training-induced inflammation and ferroptosis via Nrf2/FTH1/GPX4 pathway in intestine of mice

**DOI:** 10.55730/1300-0144.5604

**Published:** 2023-03-03

**Authors:** Zhe XU, Xiaonan SUN, Bin DING, Ming ZI, Yan MA

**Affiliations:** Department of Physical Education, Heilongjiang Bayi Agricultural University, Daqing, China

**Keywords:** Resveratrol, high intensity exercise training, ferroptosis, inflammation, Nrf2/FTH1/GPX4 pathway

## Abstract

**Background/aim:**

Moderate exercise has beneficial effects for human health and is helpful for the protection against several diseases. However, high intensity exercise training caused gastrointestinal syndrome. Resveratrol, a plant extract, plays a vital role in protecting various organs. However, whether resveratrol protected mice against high intensity exercise training-induced intestinal damage remains unclear. In this study, our objective was to investigate the protective effects and mechanism of resveratrol in high intensity exercise training-treated mice.

**Materials and methods:**

Mice were treated with swimming exercise protocol and/or resveratrol (15 mg/kg/day) for 28 consecutive days. Then, the mice were sacrificed, and a series of evaluation indicators, including inflammatory factors and intestinal permeability of the gut, were measured based on this model. The expressions of inflammatory factors (tumor necrosis factor (TNF)-α; interferon (IFN)-γ, interleukin (IL)-6 and IL-10), oxidative stress (Nrf2, glutathione (GSH), hydrogen peroxide (H_2_O_2_), catalase (CAT) and malondialdehyde (MDA)), intestinal barrier (gut permeability, ZO-1, Occludin and Claudin-1 as well as ferroptosis (Fe^2+^, Fe^3+^, SLC7A11, glutathione peroxidase 4 (GPX4) and ferritin heavy chain 1 (FTH1)) were measured, respectively.

**Results:**

High intensity exercise training induced colon damage, manifested as inflammation (increased TNF-α, IFN-γ and IL-6 concentrations, and decreased IL-10 concentration), oxidative stress (the increase of H_2_O_2_ and MDA concentration, and the reduced CAT and GSH activities), intestinal barrier injury (increased gut permeability and intestinal fatty-acid binding protein concentration, and inhibited ZO-1, Occludin and Claudin-1 expressions) and ferroptosis (the increased of Fe^2+^ and Fe^3+^ concentrations, and suppressed phosphorylated Nrf2, SLC7A11, GPX4 and FTH1), which was relieved by resveratrol treatment in mice.

**Conclusion:**

Resveratrol attenuated high intensity exercise training-induced inflammation and ferroptosis through activating Nrf2/FTH1/GPX4 pathway in mouse colon, which providing new ideas for the prevention and treatment of occupational disease in athlete.

## 1. Introduction

Regular, nonexhaustive and moderate physical exercise (3.0–5.9 metabolic equivalents) has beneficial effects for not only human health in healthy populations, but protects against several diseases (e.g., obesity, colon cancer, cholelithiasis, constipation and cardiovascular diseases) [1]. However, acute strenuous exercise causes the disorder, dysfunction, or even injury of certain tissues and organs, especially of the gastrointestinal tract [2]. The prolonged and/or high intensity exercise training/exhaustive exercise (e.g., marathon, cycling and triathlon) would cause gastrointestinal syndrome (GIS), manifested with heartburn, nausea, vomiting, diarrhea, cramping, gastric pain, and even gastrointestinal bleeding, resulting in the reduction of the exercise performance in training and competitive events [3,4].

The intestinal mucosa is composed of a simple columnar epithelium, surface mucus layers, and underlying immune cell containing lamina propria [5]. The intestinal epithelium not only selects absorption of nutrients, water, and electrolytes, but also forms a dynamic physical barrier through tight junction proteins (e.g., claudins, occludin, and zona occludens) that separates mucosal tissues from luminal commensal bacteria, pathogens, and dietary antigens [6,7]. Precise regulation of epithelial barrier function is therefore required for maintaining mucosal homeostasis and depends, in part, on barrier-forming elements within the epithelium and a balance between pro- and antiinflammatory factors (tumor necrosis factor-α, TNF-α; interferon-γ, IFN-γ; interleukin-6, IL-6 and IL-10) in the mucosa [8]. In addition, intestinal fatty-acid binding protein (I-FABP) is expressed in epithelial cells of the mucosal layer of the small intestine tissue. When intestinal mucosal damage occurs, I-FABP is released into the circulation and its plasma concentration increases. Thus, I-FABP is a biomarker of intestinal barrier dysfunction [8].

Ferroptosis is a form of regulated cell death characterized by the iron-dependent accumulation of lipid hydroperoxides to lethal levels [9]. Excessive iron regulates ferroptosis by producing lethal reactive oxygen species (ROS) via the Fenton reaction. In addition, cysteine, produced from dipeptide cystine, is transported into the cell through the cell surface cystine/glutamate antiporter system Xc^−^ (composed of SLC3A2 and SLC7A11 dimers), and then promote the synthesis of glutathione (GSH) and glutathione peroxidase 4 (GPX4) to inhibit ferroptotic cell death [10]. Ferroptosis participates in multiple intestinal diseases, including intestinal ischaemia/reperfusion injury, inflammatory bowel disease and colorectal cancer [11]. Blocking the ferroptotic process alleviated dextran sulfate sodium-induced colitis, and ferroptosis limited the migration, invasion and proliferation of colorectal cancer cells [12,13]. However, whether ferroptosis is involved in GIS remains unclear.

Resveratrol (trans-3,4,5-trihydroxystilbene) the phenolic substance isolated initially from Veratrum grandiflorum in 1939 and richly present in red wine, rhubarb, peanuts, soy and fruits (e.g., blueberries, many red grape varieties and peanuts to name a few) [14]. Resveratrol has antiinflammatory and immunoregulatory functions, with the advantages of low price and few side effects, attracting attention of scientists and medical doctors for many decades [15]. Supplemental resveratrol is beneficial to human health and could ameliorate several diseases, including cancer, cardiovascular disease, ischemic injury and inflammatory bowel diseases [16–18]. However, whether resveratrol attenuate GIS induced by high intensity exercise training remains unclear. Therefore, the present study was conducted to determine the protective effects and mechanism of resveratrol on high intensity exercise training-induced colon injury in mice.

## 2. Materials and methods

### 2.1. Animals and experimental protocol

Kunming mice (8 weeks old), weighed 20–22 g, were housed in the Biomedical Research Center, Heilongjiang Bayi Agricultural University. The housing conditions were maintained at a constant temperature (24 ± 2 °C), relative humidity at 55 ± 5%, and on a 12-h light/12-h dark cycle. Throughout the experiment, chips were replaced every 3 days and all mice had free access to water and food. Forty mice, 10 each, were randomly divided into nonexercise (NE) group (ethanol intragastric administration), high intensity exercise training (EE) group (ethanol intragastric administration+exercise training), resveratrol (RES) group (25 mg/kg/day resveratrol intragastric administration) and resveratrol + high intensity exercise training (RES+EE) group (25 mg/kg/day resveratrol intragastric administration+exercise training). Mice were administrated ethanol or resveratrol at a dose of (25 mg/kg/day; Cayman Chemicals, MI, USA, catalog no. NC9382296) by oral gavage for 28 consecutive days [19]. At the end of the 28 days, the mice were made to swim to exhaustion with a load corresponding to 5% of their body weight in the form of steel rings attached to the tail root in a tank (30 × 30 × 40 cm) filled with warm water and to a depth of 25 cm for 4 weeks. The high intensity exercise training was defined as the inability to raise its face to the water surface within 5 s. Seven mice from each group were sacrificed immediately, and the other three mice were used for gut permeability experiment. The serum and colon tissues were removed and stored at −80 °C. All animal experiments were approved by the Ethics Committee on the Use and Care of Animals, Heilongjiang Bayi Agricultural University, China.

### 2.2. Gut permeability assay

Mice were orally administered with a fluorescein isothiocyanate (FITC)-dextran (40,000 kDa; Sigma-Aldrich, catalog no. 46944, 125 mg/mL) at a dose of 600 mg/kg body weight. The blood samples were collected 2 h later and then were centrifuged at 6000 × g for 10 min at 4 °C. The serum was diluted with the equal volume of phosphate-buffered saline (PBS) and analyzed using a SpectraMax i3x Multi-Mode Microplate Reader (Molecular Devices, San Jose, CA) with an excitation wavelength at 485 nm and an emission wavelength at 535 nm. The standard curve was applied for calculating the concentration of FITC-dextran through a serial dilutions of FITC-dextran in PBS according to the manufacturers’ instructions.

### 2.3. Serum IFN-γ, IL-6, IL-10, TNF-α and I-FABP concentrations analysis

Serum was used to detect IFN-γ, IL-6, IL-10, TNF-α and I-FABP concentrations using ELISA kits (Shanghai Enzymatic Biotechnology Co., Ltd, Shanghai, China, catalog no. ml002277, ml063159, ml037873, ml002095 and ml037857) according to the manufacturers’ instructions. There were three replicates in each group. Each sample was assayed in duplicate, and IFN-γ, IL-6, IL-10, TNF-α and I-FABP concentrations were derived from a standard curve composed of serial dilutions (25–80, 3.75–120, 12.5–400, 20–640 and 0–800 pg/mL).

### 2.4. ZO-1, Occludin and Claudin-1 mRNA expressions analysis

ZO-1, Occludin and Claudin-1 mRNA expressions were analyzed by quantitative real-time reverse transcription-polymerase chain reaction (qRT-PCR). Total RNA was extracted from the mouse colon using Trizol Reagent (Invitrogen Life Technologies, Grand Island, NY, catalog no. 15596026), and total RNA was reverse transcribed using TransScript First-Strand complementary DNA (cDNA) Synthesis SuperMix (TransGen Biotech, Beijing, China, catalog no. AT301), according to the manufacturer’s instructions. The PCR primers were designed according to the NCBI database (Table). PCR was performed in a StepOnePlus Real-Time PCR System (Oxoid, Thermo Fisher Scientific, MA, USA) using a SYBR Premix Ex Taq (Takara Bio, Japan, catalog no. RR42LR). Each sample was examined in triplicate, and normalized to β-actin with the 2^−ΔΔCT^ method [20].

### 2.5. Fe^2+^, Fe^3+^, GSH, H_2_O_2_ and MDA concentrations, and CAT activities analysis

Mouse colon was used to detect Fe^2+^, Fe^3+^, GSH, H_2_O_2_ and MDA concentrations, and CAT activities using ELISA kits (Dojindo China Co., Ltd, Shanghai, China, catalog no. I291; Shanghai Enzymatic Biotechnology Co., Ltd, Shanghai, China, catalog no. A006, A007 and A003) according to the manufacturers’ instructions. There were three replicates in each group and each sample was assayed in duplicate.

### 2.6. Western blot analysis

Mouse colon was lysed in RIPA, with 1 mM PMSF, protease inhibitor cocktail and phosphatase inhibitor and protein concentration quantified using a BCA Protein Assay kit, according to manufacturer’s instructions. Proteins (30–100 μg) were separated by 12% sodium dodecyl sulfate-polyacrylamide gels electrophoresis (SDS-PAGE) and electrotransferred to PVDF membranes (Millipore, USA) by a wet transferor (BIO-RAD, USA). Then the PVDF membranes were blocked at room temperature for 2 h in 5% nonfat dry milk in 0.1% Tween-20-Tris buffered saline, pH 7.4 (TBST). After blocking, membranes were incubated with specific primary antibodies of phosphorylation Nrf2 (Ser40, P-Nrf2, GeneTex, catalog no. GTX02873), Nrf2, SLC7A11, GPX4, FTH1 and GAPDH (Cell Signaling Technology, catalog no.12721, 98051, 59735, 4393 and 5174) at 4 °C overnight. Thereafter, membranes were probed with HRP-conjugated secondary antibody for 1 h at room temperature and fluorescence detected with an enhanced chemiluminescence system (ECL). Results were normalized to GAPDH, and band density was analyzed with ImageJ (National Institutes of Mental Health, Bethesda, MD, USA) [20].

### 2.7. Statistical analysis

Effects of resveratrol on high intensity exercise training-induced intestinal mucosal barrier dysfunction were determined with one-way analysis of variance, with an LSD test used to detect differences between groups. All statistical analyses were done with SPSS 23.0 (IBM Corporation, Armonk, NY, USA) and histograms generated with Graphpad Prism 7.0 (GraphPad Software, Inc., San Diego, CA, USA). Data were expressed as mean ± standard deviation (SD), with *p* < 0.05 and *p* < 0.01 considered significant and highly significant, respectively.

## 3. Results

### 3.1. Resveratrol relieved high intensity exercise training-induced inflammation in mouse serum

**Figure 1:**
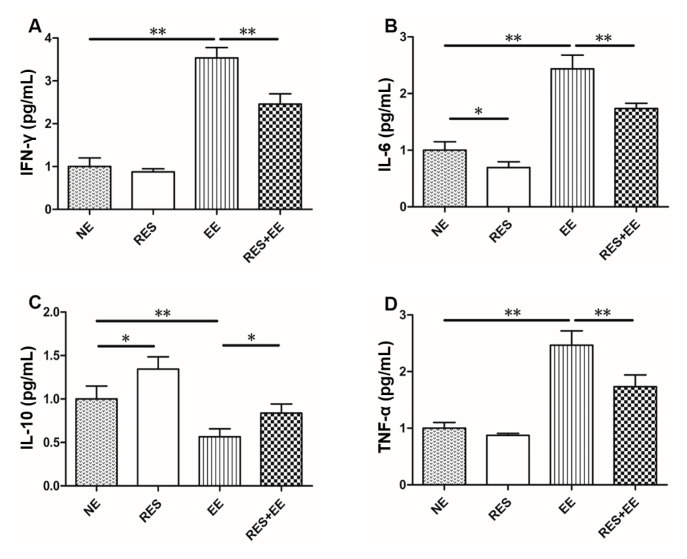
Protective effects of RES on EE-induced IFN-γ, IL-6, IL10 and TNF-α release in mice. Data represent means ± SD of 3 independent experiments with similar results. NE: nonexercise group, EE: high intensity exercise training, RES: resveratrol group, RES+EE: resveratrol+high intensity exercise training. * *p* < 0.05, ** *p* < 0.01

High intensity exercise training increased (*p* < 0.01) serum IFN-γ (Figure 1A), IL-6 (Figure 1B), and TNF-α (Figure 1C) concentrations, and inhibited (*p* < 0.01) IL-10 release (Figure 1D), which was attenuated (*p* < 0.05) by resveratrol treatment in mice. In addition, resveratrol increased (*p* < 0.05) IL-10 concentrations in EE group.

### 3.2. Resveratrol inhibited increase of gut permeability induced by high intensity exercise training in mouse colon

**Figure 2:**
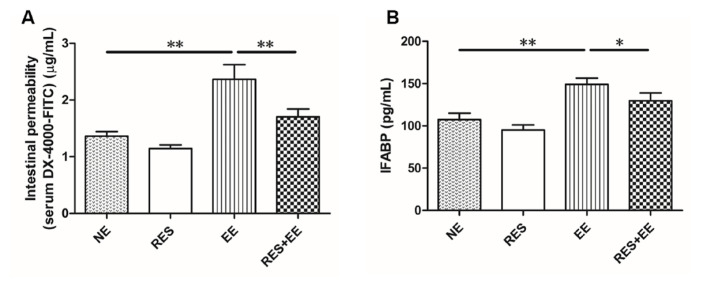
The effects of RES on intestinal permeability and IFABP concentrations in EE-treated mice. Data represent means ± SD of 3 independent experiments with similar results. NE: nonexercise group, EE: high intensity exercise training, RES: resveratrol group, RES+EE: resveratrol+high intensity exercise training. * *p* < 0.05, ** *p* < 0.01.

High intensity exercise training increased (*p* < 0.01) intestinal permeability (serum DX-4000-FITC; Figure 2A) and serum I-FABP concentrations (Figure 2B), which was relieved (*p* < 0.05) by resveratrol treatment in the mouse colon.

### 3.3. Resveratrol alleviated the disrupted intestinal barrier integrity in high intensity exercise training-treated mouse colon

**Figure 3:**
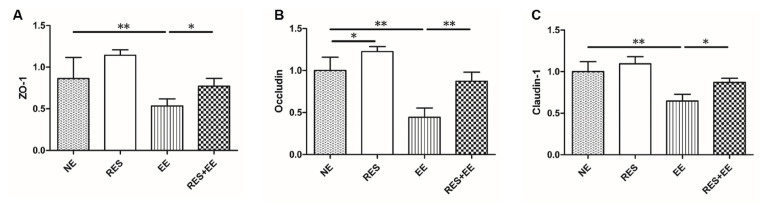
The effects of RES on ZO-1, Occludin and Claudin-1 mRNA expressions in EE-treated mice. Data represent means ± SD of 3 independent experiments with similar results. NE: nonexercise group, EE: high intensity exercise training, RES: resveratrol group, RES+EE: resveratrol+high intensity exercise training. * *p* < 0.05, ** *p* < 0.01.

The mRNA levels of ZO-1 (Figure 3A), Occludin (Figure 3B) and Claudin-1 (Figure 3C) were decreased (*p* < 0.01) by high intensity exercise training, which was alleviated (*p* < 0.05) by resveratrol treatment in the mouse colon. In addition, resveratrol increased (*p* < 0.05) Occludin mRNA expressions in EE group.

### 3.4. Resveratrol suppressed the oxidative stress in high intensity exercise training-treated mouse colon

**Figure 4:**
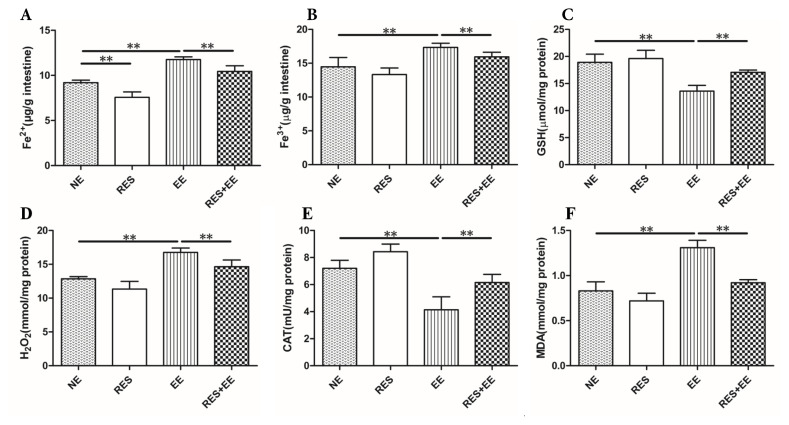
The effects of RES on Fe^2+^, Fe^3+^, GSH, H_2_O_2_ and MDA concentrations, and CAT activities in EE-treated mice. Data represent means ± SD of 3 independent experiments with similar results. NE: nonexercise group, EE: high intensity exercise training, RES: resveratrol group, RES+EE: resveratrol+high intensity exercise training. ** *p* < 0.01.

The concentrations of GSH (Figure 4C), H_2_O_2_ (Figure 4D) and MDA (Figure 4F) were decreased (*p* < 0.01), and CAT activity (Figure 4E) was increased by high intensity exercise training, which was alleviated (*p* < 0.01) by resveratrol treatment in the mouse colon.

### 3.5. Resveratrol reversed the ferroptosis through Nrf2/GPX4 pathway in high intensity exercise training-treated mouse colon

Fe^2+^ and Fe^3+^ concentration (Figures 4A and 4B) were increased (*p* < 0.01) by high intensity exercise training, which was relieved (*p* < 0.01) by resveratrol treatment in the mouse colon. In addition, resveratrol decreased (*p* < 0.01) Fe^2+^ concentration in NE group.

**Figure 5:**
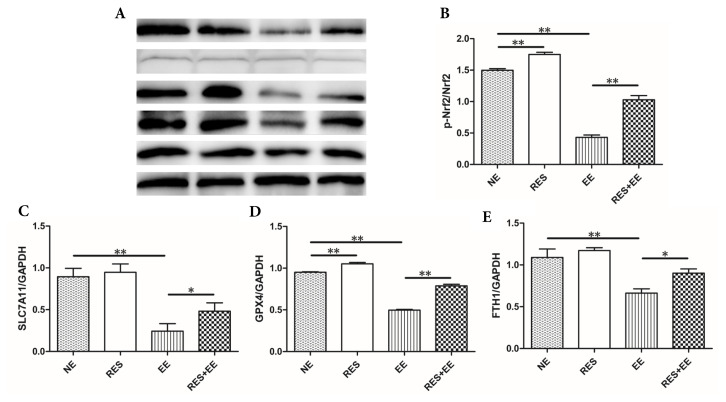
The effects of RES on ferroptosis through Nrf2/GPX4 pathway in EE-treated mice. Data represent means ± SD of 3 independent experiments with similar results. NE: nonexercise group, EE: high intensity exercise training, RES: resveratrol group, RES+EE: resveratrol+high intensity exercise training. * *p* < 0.05, ** *p* < 0.01.

The protein expressions (Figure 5) of P-Nrf2, SLC7A11, GPX4 and GAPDH were decreased (*p* < 0.01) by high intensity exercise training, which was increased (*p* < 0.05, *p* < 0.01) by resveratrol treatment in the mouse colon. In addition, resveratrol increased (*p* < 0.01) P-Nrf2 and GPX4 protein levels in NE group.

## 4. Discussion

Exercise-induced gastrointestinal syndrome (EIGS) refers to disturbances of gastrointestinal integrity and function that are common features of strenuous exercise [2]. Many athletes suffer from EIGS when they participate in the training or competitions, especially endurance sports, accompanied by the decreased exercise performance [21]. The intestinal health plays a crucial role in maintaining an organism’s healthy state. Therefore, effective treatment strategies for EIGS are the research hotspot at present. In this study, mice were treated with resveratrol and/or high intensity exercise training. We found that high intensity exercise training induced colon damage, manifested as inflammation, oxidative stress, intestinal barrier injury and ferroptosis, which was attenuated by resveratrol treatment in mice of this study, consistent with resveratrol increased expression levels of tight junction proteins, H_2_O_2_ level and Nrf2 phosphorylation expression in H_2_O_2_-treated mice [22]. Taken together, resveratrol relieved high intensity exercise training-induced inflammation and ferroptosis through Nrf2/FTH1/GPX4 pathway in mice.

Inflammation is a response triggered by damage to living tissues. INF-γ, IL-6 and TNF-α are the important proinflammatory cytokines, which can trigger cellular activation, differentiation, and recruitment. INF-γ promotes cytotoxic activity, regulates major histocompatibility complex class I and II protein expression and antigen presentation, inhibits cell growth and apoptosis and controls the extension of the immune response [23]. IL-6 is recognized to be a major modulator of local or systemic acute inflammatory responses. TNF-α is one of the most important endogenous shock factor. IL-10 is an antiinflammatory cytokine that inhibits the production of proinflammatory cytokines such as INF-γ, TNF-α, and IL-6 to modulate innate and adaptive immunity. In this study, high intensity exercise training significantly increased serum INF-γ, IL-6 and TNF-α concentrations, and significantly inhibited serum IL-10 concentration in mice, consistent with the previous research [24]. Thus, we deduced that high intensity exercise training caused inflammation in mice of this research. In addition, in this study, pretreating with resveratrol (15 mg/kg/day) significantly inhibited the increase in serum IFN-γ, IL-6 and TNF-α concentrations, and enhanced the decreased serum IL-10 release in high intensity exercise training-treated mice, consistent with resveratrol inhibiting inflammatory response (increased IFN-γ, IL-6, and TNF-α levels, and decreased IL-10 concentrations) in Toxoplasma gondii-infected mice [25]. The overall configuration of immune response in the current study was comparable to similar studies, as resveratrol inhibited LPS-induced the increase in the production of TNF-α, IL-6, IL-8 and IFN-β [26]. Thus, we inferred that resveratrol mitigated high intensity exercise training-induced inflammation in mice.

The intestinal permeability reflects the intestinal barrier function. The research reported that prolonged and strenuous physical exercise increases intestinal permeability [27]. In this study, resveratrol inhibited the increase in IFABP release and serum DX-4000-FITC concentrations induced by high intensity exercise training, which indicating that resveratrol alleviated the increased intestinal permeability induced by high intensity exercise training in the mouse colon. In addition, high-intensity exercise also increased the intestinal permeability through inducing the dysfunction of epithelial cells and disordering tight junction proteins [28]. Resveratrol prevented the H_2_O_2_- and diquat-induced decline of Occludin, Claudin-1 and ZO-1 levels in the porcine intestinal epithelial cells and the jejunal mucosa [22, 29]. In this research, resveratrol enhanced the decrease in Claudin-1, Occludin, and ZO-1 mRNA expressions in the mouse colon, consistent with pretreatment with resveratrol increasing the reduced mRNA and protein expression levels of Claudin-1, Occludin, and ZO-1 induced by oxidative stress in the porcine intestinal epithelial cells [22]. Thus, we deduced that resveratrol attenuated high intensity exercise training-induced intestinal barrier injury.

Oxidative stress, defined as the imbalance between the antioxidant systems and oxidative system causing overdose of ROS, can disrupt cellular signaling and function. Oxidative stress is implicated in a wide range of intestinal disorders and closely associated with their pathological processes [30]. In this study, high intensity exercise training significantly increased H_2_O_2_ and MDA concentrations, and significantly suppressed GSH concentrations and CAT activity, which indicating that high intensity exercise training induced oxidative stress in mouse colon.

Ferroptosis has been associated with dysfunction of the intestinal epithelium and research on ferroptosis may provide a new understanding of intestinal disease pathogenesis that benefits clinical treatment [11]. Ferroptosis is a form of iron-dependent, nonapoptotic regulated cell death, characterized by the accumulation of lethal lipid hydroperoxides and loss of the activity of the lipid repair enzyme [31]. Fe^2+^ can convert H_2_O_2_ into OH free radical (·OH) through the fenton reaction, and then produced ROS will undergo lipid peroxidation reaction with the polyunsaturated fatty acids in the biofilm, leading to ferroptosis [32]. In addition, SLC7A11, as a part of system Xc^−^, transfers extracellular cysteine to cells and converts it into cysteine for the synthesis of glutathione [33]. The selective inhibition of system Xc^−^ reduced GSH and GPX4 synthesis in cells and the accumulation of oxygen free radicals, which eventually led to cell death, thereby inhibiting ferroptosis [34]. Moreover, ferritin heavy chain 1 (FTH1), a marker of ferroptosis, regulated ferroptosis [35]. In this study, high intensity exercise training significantly increased Fe^2+^, Fe^3+^, H_2_O_2_ and MDA concentrations, and significantly suppressed GSH concentrations, CAT activity, and SLC7A11, GPX4 and FTH1 protein expressions, which indicating that high intensity exercise training induced ferroptosis in mouse colon. In addition, DAMPs are released from, or exposed on, injured or stressed cells with ferroptosis, and then induced inflammation through TLR4, AGER and STING1 pathways [36]. Thus, we deduced that high intensity exercise training caused inflammation through ferroptosis.

Resveratrol is a natural compound that can activate the Nrf2 transcription factor to prevent inflammation and oxidative stress [37]. Nrf2 is a transcription factor which was identified as a master regulator of defensive responses to oxidative stress. Under stimulatory signal, Nrf2 activation enhanced iron storage capacity and GPX4 activity by elevating FTH1 expression [38]. In this study, resveratrol significantly decreased Fe^2+^, Fe^3+^, H_2_O_2_ and MDA concentrations, and significantly promoted GSH concentrations, CAT activity, and SLC7A11, GPX4 and FTH1 protein expressions in EE group, which suggesting that resveratrol reversed high intensity exercise training-induced ferroptosis through Nrf2/FTH1/GPX4 pathway.

**Figure 6:**
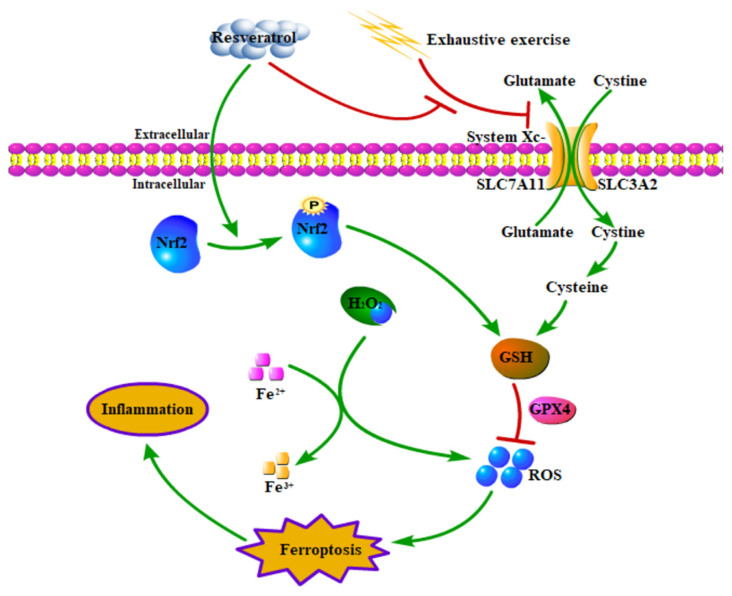
Resveratrol attenuated high intensity exercise training-induced and inflammation through Nrf2/FTH1/GPX4 pathway-regulated ferroptosis in mice. Green arrow and red bar indicate stimulation and inhibition, respectively.

In conclusion, high intensity exercise training induced inflammation and intestinal mucosal barrier dysfunction through ferroptosis, whereas resveratrol attenuated high intensity exercise training-induced inflammation through Nrf2/FTH1/GPX4 pathway-mediated ferroptosis in mice (Figure 6), which providing new preventive strategies for athletes against EIGS.

## Figures and Tables

**Table:** The primer sequence of the amplification target gene.

Gene	Upstream and downstream primer sequence
ZO-1	Forward: 5′-CTGGTGAAGTCTCGGAAAAATG-3′
	Reverse: 5′-CATCTCTTGCTGCCAAACTATC-3′
Occludin	Forward: 5′-TGCTTCATCGCTTCCTTAGTAA-3′
	Reverse: 5′-GGGTTCACTCCCATTATGTACA-3′
Claudin-1	Forward: 5′-ACGGCTCCGTTTTCTAGATGCC-3′
	Reverse: 5′-CGTTTGGCTGCTGCTCTTGC-3′
*β-*actin	Forward: 5′-CTACCTCATGAAGATCCTGACC-3′
	Reverse: 5′-CACAGCTTCTCTTTGATGTCAC-3′
